# Rapid Detection of Feline Calicivirus Using Lateral Flow Dipsticks Based on CRISPR/Cas13a System

**DOI:** 10.3390/ani14243663

**Published:** 2024-12-18

**Authors:** Zichuang Zhang, Jing Li, Chengqi Zhang, Xue Bai, Tie Zhang

**Affiliations:** 1 Key Laboratory of Special Animal Epidemic Disease, Ministry of Agriculture, Institute of Special Animal and Plant Sciences, Chinese Academy of Agriculture Sciences, Changchun 130112, China; zzc5665@163.com (Z.Z.); zcq971102@163.com (C.Z.); 2 The College of Veterinary Medicine, Hebei Agricultural University, Baoding 071001, China; lee12355355@163.com

**Keywords:** feline calicivirus, recombinase-aided amplification, CRISPR/Cas13a, lateral flow dipstick, rapid detection

## Abstract

Feline calicivirus (FCV) is one of the most common viral pathogens in domestic cats worldwide, which mainly causes upper respiratory tract infections in felines and seriously threatens the health of felines. Consequently, it is crucial to establish a rapid detection method to efficiently take control and prevent the spread of FCV. To construct the Cas13a-RAA-LFD reaction system, this study specifically designed recombinase-aided amplification (RAA) primers added with a T7 promoter and CRISPR RNA (crRNA), which were both based on the FCV relatively conserved sequence. The Cas13a protein cleaved the reporting probes only when crRNA recognized the target sequence. The results could be directly observed by lateral flow dipsticks (LFDs). To evaluate this system, factors such as RAA amplification time, Cas13a protein concentration, crRNA concentration, and CRISPR reaction time were optimized. Then, a comparison of the coincidence rate for clinical samples between this method and the polymerase chain reaction (PCR) agarose electrophoresis method was performed to evaluate the reliability of the method. Eventually, the results indicated that the target gene could be effectively amplified by the Cas13a-RAA-LFD method, and the results could be visually observed by LFD. The method could detect FCV specifically, whilst having no cross-reaction with other common viruses which infect felines, such as feline parvovirus (FPV), feline coronavirus (FCoV) and feline herpesvirus (FHV). This method is extremely sensitive and has been validated to detect viral nucleic acids down to 10^0^ copies/μL. The good reproducibility and stability of the method were also verified by this study. Testing of clinical samples proved that the coincidence rate of clinical detection reached 96.39%. In summary, this study established a simplistic, efficient, accurate, and visualized FCV detection method, which can be utilized for early prevention and control of FCV.

## Simple Summary

Feline calicivirus (FCV) is widely prevalent in domestic cats around the world. As an RNA virus, FCV is highly mutable and prevalent. The spread of the virus mainly causes upper respiratory tract infections by low pathogenic FCV, or even, leads to death by virulent systemic FCV, which poses a serious threat to felines. Currently, there is no specific drug for this virus. Efficacious FCV vaccines protect against severe disease but not against infection. What is more, due to the variability of the ORF2 of the virus, the mutation of FCV happens at high frequencies during transportation, which result in the low cross-protection of the vaccine. And the vaccine is ineffective which can only alleviate some of the clinical symptoms, especially in some virulent systemic FCV infection cases. Consequently, there is an urgent need to develop a method that allows rapid detection of the virus for control and prevention of FCV. In this study, we designed the CRISPR/Cas13a-LFD reaction system and evaluated its specificity, sensitivity, reproducibility, and clinical reliability after establishing and optimizing, which provides an efficient tool for the early diagnosis of FCV.

## 1. Introduction

Feline calicivirus (FCV) belongs to the genus Calicivirus of the family Caliciviridae and is a highly mutagenic RNA virus that infects all felines and is one of the most common viral pathogens in domestic cats worldwide [1]. The virus can cause upper respiratory tract infection in felines, mainly manifested as conjunctivitis, stomatitis, tracheitis, and bronchitis, accompanied by biphasic fever, and sometimes lead to lameness, abortion, oral ulcers, and other diseases [2,3]. FCV encodes three open reading frames (ORF). ORF1 encodes non-structural proteins. ORF2 and ORF3 are located at the 3′ end of the genome, encoding capsid proteins and structural proteins, respectively [4]. FCV is mainly transmitted through the air and direct contact. For example, secretions from coughs, sneezes, saliva, and the nose may contain pathogenic agents. Moreover, FCV can also be transmitted through a contaminated food or water source [5]. The virus is infectious for up to a month on dry surfaces at room temperature and even longer in colder environments [6]. The transmission of FCV is a serious threat to the health of felines. Currently, there is no specific drug for the treatment of the virus. Efficacious FCV vaccines protect against severe disease but not against infection [7]. What is more, due to the variability of the ORF2 of the virus, the mutation of FCV happens at high frequencies during transportation, which result in the low cross-protection of the vaccine. And the vaccine is ineffective which can only alleviate some of the clinical symptoms, especially in some virulent systemic FCV infection cases [8]. Consequently, detecting the virus early and rapidly is crucial for the prevention and control of FCV.

**Figure 1:**
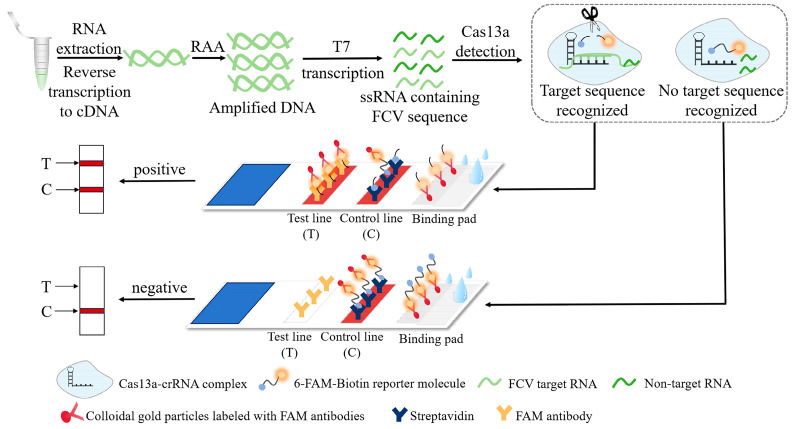
Principle of Cas13a-RAA-LFD.

Clustered regularly interspaced short palindromic repeats (CRISPR) and its related proteins (Cas) serve as an adaptive immune system to protect prokaryotes from foreign nucleic acids, such as viruses and plasmids [9]. A class of CRISPR/Cas systems includes type I, type III, and type IV [10]. Cas13a in the type IV system is an RNA-guided ribonuclease with collateral cleavage activity. When combined with CRISPR RNA (crRNA), this crRNA can specifically complement the target sequence, cut a large number of labeled single-stranded DNA or RNA, and enable amplification of the signal while providing additional sensitivity [11,12]. Recombinase add amplification (RAA) is one of the rapid amplification technology which has emerged in recent years, which mainly consists of three components: DNA polymerase, recombinase, and single-chain binding protein [13], which can replace thermal cycles and achieve high-speed amplification at low temperature [14]. The combination of the RAA technology and the CRISPR/Cas13a system can amplify the initial signal, make pathogen detection more convenient and efficient, and provide the possibility of field detection [15,16]. The principle is that after the amplification of the pathogen, the target DNA was transcribed into RNA through the T7 in vitro transcription system. When the target sequence exists, the target RNA specifically binds to the crRNA-Cas13a protein complex, activating the “collateral cleavage” activity of the Cas13a protein, which cleaves the reporter molecule. And then, the compound of the reporter molecule and colloidal gold particles labeled with the FAM antibody were bound by the secondary antibody, and the T line appeared as a positive. When target sequence was not present, Cas13a was not activated and did not perform the cleavage function, so the compound of the reported molecule and colloidal gold particles with FAM antibodies was captured by streptavidin, and the control line appeared as a negative (Figure 1). Up to the present, many studies have combined RAA technology with the CRISPR/Cas13a system to achieve the goal of rapid detection, such as African swine fever virus detection [17], Helicobacter pylori detection [18], norovirus detection [19] and so on.

This study combined the RAA and the CRISPR/Cas13a methods with the lateral flow dipstick (LFD) method. According to the FCV ORF1 gene uploads in GenBank, a relatively conserved sequence was selected and the crRNA and RAA primer were designed. To establish and optimize the system, the qualities of the method were evaluated, such as specificity, sensitivity, repeatability, and clinical reliability. A fast, sensitive, specific, and visual FCV detection method was established, which provided an efficient tool for the early diagnosis of FCV.

## 2. Materials and Methods

### 2.1. Extraction of Nucleic Acid

Feline calicivirus (FCV), feline parvovirus (FPV, GenBank number: PP973493.1), feline coronavirus (FCoV), and feline herpesvirus (FHV) were all detected and stored in the Key Laboratory of Special Animal Epidemic Disease, Ministry of Agriculture. Extracting the DNA of FPV and FHV and the RNA of FCV and FCoV (TIANamp Virus DNA/RNA Kit, Tianjin, China), and then reverse-transcribing the RNA of FCV and FCoV to cDNA and carefully storing it at −20 °C.

### 2.2. Design of Primers and crRNA, Selecting of Primers

**Figure 2:**
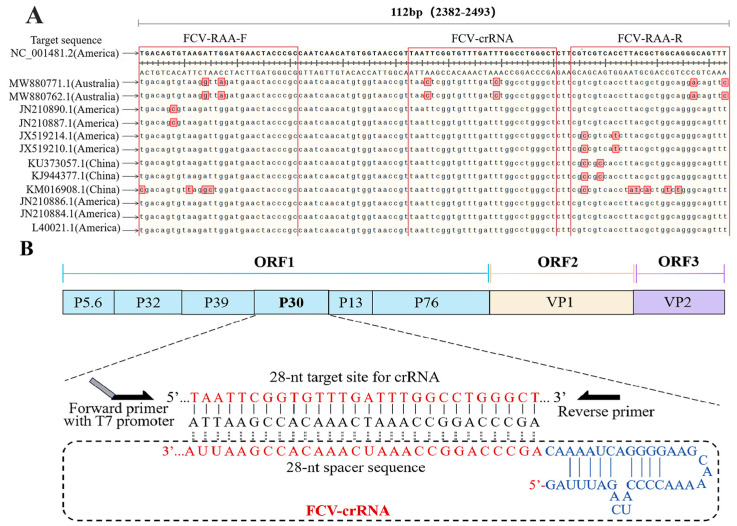
Primer and crRNA design. (**A**). Primer and crRNA design. Nucleobases in red are different bases in the comparison sequence. (**B**). Sequence comparison.

According to the complete FCV gene sequences registered in GenBank (entry number: NC_001481.2, MW880771.1, MW880762.1, JN210890.1, JN210887.1, JX519214.1, JX519210.1, KU373057.1, KJ944377.1, KM016908.1, JN210886.1, JN210884.1, and L40021.1), comparative analysis was performed using SnapGene 6.0.2 and DNAMAN 9.0.1.116 software (Figure 2A). According to the previous research [20] and comparing the sequences, the conserved sequence of the ORF1 P30 gene segment was determined, and 3 pairs of specific RAA primers were designed. The T7 RNA polymerase promoter sequence was added to the 5′ end of the forward primer. In addition, crRNA was designed in the conserved sequence of the P30 gene, consisting of LwaCas13a and a 28 nt length of target sequence, that was included in the primer amplification region (Figure 2B). Primers crRNA in vitro transcription template (crRNA-IVT) and T7-3G oligonucleotides (Table 1) were synthesized by a biotechnology company (Sangon Bioengineering Co., Ltd., Shanghai, China).

By using FCV cDNA as a template, a RAA amplification reaction was performed by a Nucleic Acid Test Strip Rapid Test Kit (Anhui Microanaly Gene Technology Co., Ltd., Anhui, China). The reaction system was 50 μL, which consisted of; A buffer 32.9 μL, purified water 8.6 μL, B buffer 2.5 μL, forward and reverse primer 2 μL (10 µM) each, and the cDNA template 2 μL. The amplified products were purified after performing a reaction at 37 °C for 30 min. A 2% agarose electrophoresis method was then used to observe results and select optimal primers pairs.

### 2.3. Transcription and Purification of crRNA

The processes were as follows: 10 µL of reaction system was mixed, which included ultra-pure water 7 µL, crRNA-IVT 1 µL (100 µM), T7-3G oligonucleotide 1 µL (100 µM), and Standard Taq buffer (10×) 1 µL. After denaturing it in PCR apparatus at 95 °C for 5 min, slowly cooling it down to 4 °C by 0.1 °C/s. After the reaction, the 10 μL reaction product was mixed with 10 μL NTP buffer mixture, 2 μL T7 RNA polymerase mixture, and 18 μL ultra-pure water, transcribed at 37 °C overnight, and slowly cooled down to 4 °C at 0.1 °C/s. After in vitro transcription, crRNA was purified using a Spin Column RNA Cleanup and Concentration Kit (Sangon Bioengineering Co., Ltd., Shanghai, China), the concentration was measured, and saved at −80 °C.

### 2.4. Construction of Standard Plasmid

The PCR amplification was performed using cDNA of FCV as the template. The PCR reaction system (25 μL) consisted of 2×Taq Mix (12.5 μL), ddH_2_O (10.5 μL), forward primer and reverse primer (10 μM, 0.5 μL) each, and the template (1 μL). The reaction procedure was as follows: the sample was initiated at 94 °C for 5 min, followed by 35 cycles at 94 °C for 30 s, 50 °C for 30 s, 72 °C for 30 s, and then extended at 72 °C for 5 min. A 2% agarose gel electrophoresis method was then used to observe the results and verify the PCR products. And then, the gels were cut and recovered by a SanPrep Column DNA Gel Extraction Kit (Sangon Bioengineering Co., Ltd., Shanghai, China). Before introducing the plasmids into DH5α competent cells, the recovered DNA was connected to a T-Vector pMD 20 vector. White colonies were selected and plasmids were extracted by a plasmid extraction kit (Tiangen Biochemical Technology Co., Ltd., Beijing, China) after spending overnight to culture. The sequences of standard plasmids were detected by a biotechnology company.

Per unit volume plasmid containing DNA copies was calculated by the formula as follows:

Plasmid copy number (copies/μL) = [plasmid concentration (g/μL) × 10^−n^ × 6.02 × 10^23^]/{[Vector length (bp) + Fragment length (bp)] × 660 g/mol}

The standard plasmid was diluted 10 times by using ultra-pure water. The diluted concentrations ranged from 1 × 10^9^ to 1 × 10^0^ copies/μL in 10 gradients and the plasmids were stored at −20 °C for use.

### 2.5. Establishment of Cas13a-RAA-LFD for FCV Detection

The RAA reaction was conducted under the guidelines of the DNA isothermal amplification reaction kit (Suzhou JIENNUO Bio-Medical Technology Co., Ltd., Suzhou, China). A buffer 32.9 μL, purified water 8.6 μL, B buffer 2.5 μL, forward and reverse primer 2 μL (10 µM) each, and a cDNA template 2 μL constituted the RAA reaction system of 50 μL. The processes were as followed: the reaction reagents were added to the reaction unit tube containing dry powder, the tube cap was covered, the mixture was inverted and mixed thoroughly 8–10 times, centrifuged at low speed for 10 s, and then placed in a thermostatic environment for amplification (37 °C, 30 min).

For the Cas13a-RAA-LFD reaction system (50 μL), we used enzyme-free sterile water (28.5 μL), Cas13a (Meige Biological Technology Co. Ltd., Guangzhou, China) (80 nmol/L, 4 μL), NTP buffer mix (25 mmol/L, 4 μL), RNase inhibitor (40 U/μL, 2 μL), crRNA (80 ng/uL, 2 μL), RNA double-labeled probe (100 μmol/L, 2 μL), T7 RNA polymerase mix (5000 U/mL, 1 μL), HEPES buffer (1 mol/L, 1 μL), MgCl_2_ (1 mol/L, 0.5 μL), and the RAA amplification product (5 μL). We completely mixed the reactants, with it reacting at 37 °C, and then waited for 25 min. Subsequently, we added the mixture (50 μL) to LFD, and within 3 to 5 min the results could be observed.

### 2.6. Optimization of Cas13a-RAA-LFD for FCV Detection

A series of factors were optimized to improve the amplification efficiency. The RAA amplification time was optimized by starting at 10 min and increasing 5 min in every gradient. The appearance of a clear detection line was selected as the criterion to indicate the optimal RAA reaction time. We chose to start at 10 ng/μL and increased by 10 ng/μL in every gradient to evaluate the optimal crRNA concentration. By remaining the rest of factors unchanged, the appearance of a clear line was used as the standard to optimize the crRNA concentration. After optimization of the crRNA concentration, the concentration of Cas13a was optimized by starting at 40 nmol/L and increasing 10 nmol/L in every gradient. Starting at 10 min and increasing 5 min in every gradient to optimize the CRISPR reaction time until the appearance of clear detection line, which was under above-mentioned concentration of crRNA and Cas13a.

### 2.7. Specific Detection

To evaluate the specificity of the Cas13a-RAA-LFD, the DNA of FPV and FHV, and the cDNA of FCV, FCoV was used as a template and ddH_2_O was set as the negative control.

### 2.8. Sensitivity Detection

The diluted FCV standard plasmid (10^7^–10^0^ copies/μL) were used as templates, and ddH_2_O was set as the negative control. By repeating each method (the PCR-Agar-gel electrophoresis method, qPCR method, and Cas13a-RAA-LFD) three times, respectively, the sensitivity of the method was evaluated.

A 2×Taq Mix (12.5 μL), ddH_2_O (10.5 μL), a forward and reverse primer (10 μM, 0.5 μL) each, and a template (10^7^–10^0^ copies/μL, 1 μL) constituted the PCR reaction system (25 μL). The reaction procedure was initiated at 94 °C for 5 min, followed by 35 cycles at 94 °C for 30 s, 50 °C for 30 s, 72 °C for 30 s, and then extended at 72 °C for 5 min. By using the 2% agarose gel electrophoresis method, the PCR products were verified.

A 2×Universal Blue SYBR Green qPCR Master Mix 10.0 μL (Servicebio Technology Co., Ltd., Wuhan, China), ddH_2_O 8.6 μL, forward and reverse primers (10 μM) 0.4 μL each, and templates (10^6^–10^0^ copies/μL) 1.0 μL constituted the qPCR reaction system of 20 μL. The reaction procedure initiated at 95 °C for 30 s, followed by 40 cycles at 95 °C for 15 s, 60 °C for 30 s, and then the results could be observed.

The Cas13a-RAA-LFD used 10^5^–10^0^ copies/μL standard plasmids which were performed as templates.

### 2.9. Repeatability Detection

The repeatability of the Cas13a-RAA-LFD method was evaluated by using 3 different concentrations of FCV standard plasmids (10^6^ copies/μL, 10^3^ copies/μL, and 10^0^ copies/μL) after dilution as templates, repeating the experiment 3 times for each concentration, and setting ddH_2_O as a negative control.

### 2.10. Testing of Clinical Samples

Eye, nose, and mouth swabs of 83 cats suspected to be infected with FCV were detected by a PCR and the Cas13a-RAA-LFD, and the calculation of the coincidence rate between the two methods for clinical samples was based on the PCR method [Coincidence rate (%) = (number of all positive samples + number of all negative samples in the two test methods compared)/total number of samples × 100%].

## 3. Results

### 3.1. Preparation of Standard Plasmid

The recombinant plasmid was sequenced and compared. The concentration of the standard plasmid was measured to be 102.5 μg/mL after confirming that it was the target sequence, and the standard plasmid copy number was 3.15 × 10^10^ copies/μL.

### 3.2. Selecting of RAA Primers

**Figure 3:**
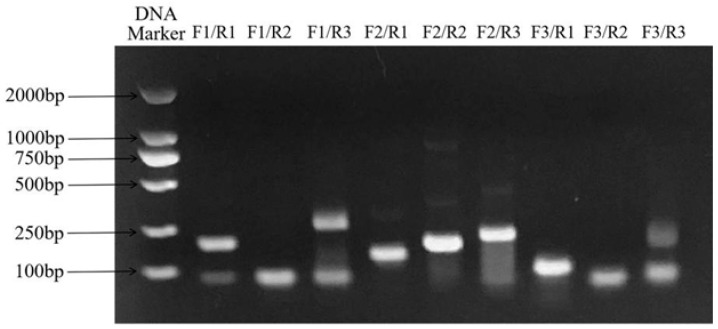
Primers selected for FCV detection by the RAA method.

The results showed that the combinations of FCV-RAA-F2/R1, F2/R2, F2/R3, and F3/R1 had clear bands and higher amplification efficiency than other primer combinations. Among them, the combination of F3/R1 had a single band and did not show non-specific amplification. Therefore, the combination of FCV-RAA-F3/R1 was the optimal primer pair. The results are shown in Figure 3.

### 3.3. Optimal Conditions of Cas13a-RAA-LFD for FCV Detection

**Figure 4:**
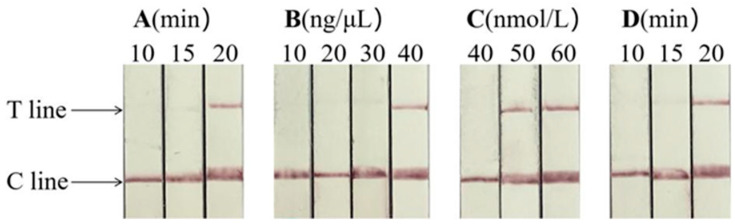
Optimal conditions of Cas13a-RAA-LFD for FCV detection (**A**). Optimization of RAA reaction time. (**B**). Optimization of crRNA concentration. (**C**). Optimization of crRNA concentration. (**D**). Optimization of CRISPR response time. C line: quality control line. T line: test line.

By using the appearance of a clear T line on dipsticks as the criterion for the test, RAA reaction time, crRNA concentration, and CRISPR reaction time were verified as 20 min, 40 ng/μL, and 20 min, respectively. The optimal concentration of Cas13a is 50 nmol/L because of the occurrence of clear detection lines existing both in 50 nmol/L and 60 nmol/L (Figure 4).

### 3.4. Specific Detection of Cas13a-RAA-LFD for FCV Detection

**Figure 5:**
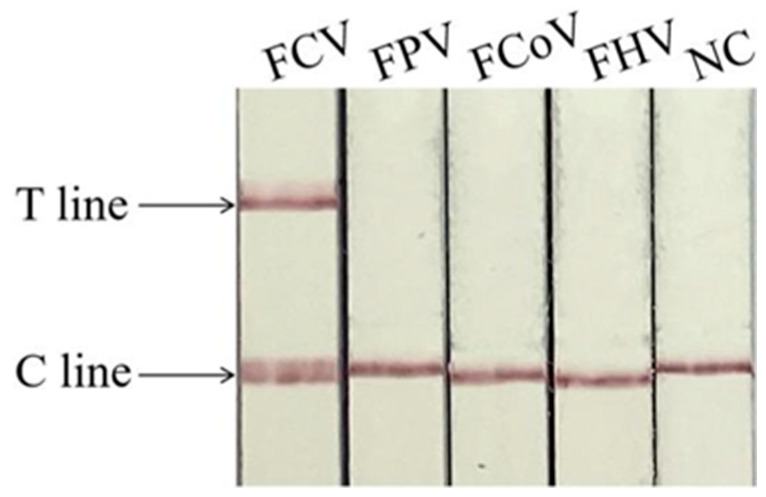
Specificity of Cas13a-RAA-LFD assay. C line: quality control line. T line: test line.

Figure 5 shows that only in FCV appears the T line, indicating that the methods for detecting FCV have no cross-reaction with detecting FPV, FCoV, and FHV, and have good specificity.

### 3.5. Sensitivity Detection of Cas13a-RAA-LFD for FCV Detection

**Figure 6:**
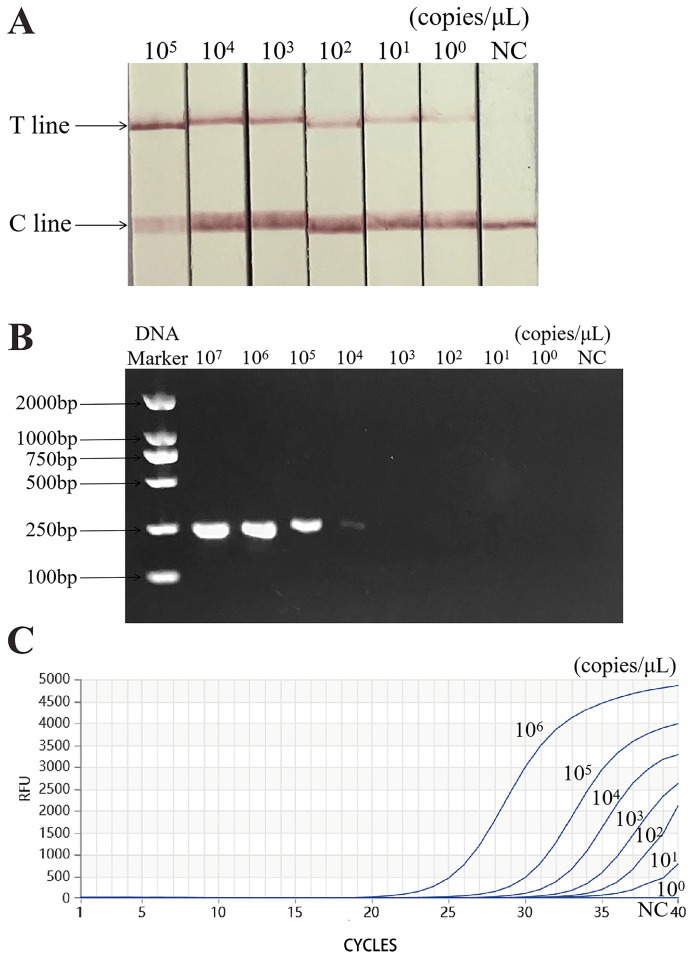
Sensitivity of Cas13a-RAA-LFD assay. (**A**). LOD of the Cas13a-RAA-LFD is 10^0^ copies/μL. (**B**). LOD of a PCR is 10^4^ copies/μL. (**C**). LOD of a qPCR is 10^1^ copies/μL. C line: quality control line. T line: test line.

Figure 6 verifies that the limit of detection (LOD) of a PCR was 10^4^ copies/μL, that of a qPCR was 10^1^ copies/μL, and that of the Cas13a-RAA-LFD it was 10^0^ copies/μL. The sensitivity of the Cas13a-RAA-LFD method is 10 times higher than that of a qPCR and 10,000 times higher than that of a PCR.

### 3.6. Repeatability Detection of Cas13a-RAA-LFD for FCV Detection

**Figure 7:**
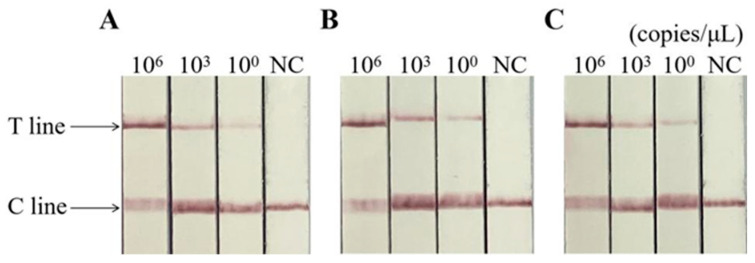
Repeatability of Cas13a-RAA-LFD assay. (**A**–**C**) are triple repeatability tests. C line: quality control line. T line: test line.

Figure 7 shows that the different concentrations of standard plasmids were all positive. In contrast, the control group were all negative, indicating that the detection method had good stability.

### 3.7. Clinical Sample Testing

Table 2 shows that 46 positive samples were detected by the Cas13a-RAA-LFD. Compared with the PCR-Agarose electrophoresis, the coincidence rate of the Cas13a-RAA-LFD was 96.39%, indicating that the method has strong detection ability and is suitable for detecting clinical samples.

## 4. Discussion

FCV is one of the most important pathogens causing infectious respiratory diseases in cats. A highly virulent FCV infection can lead to a mortality rate of up to 40–60%. Asymptomatic carriers and clinically recovered cats can still shed in the external environment for a long time, making it more difficult to prevent the spread of FCV [21].

Up to the present, the normal FCV detection methods in the clinical field include a PCR, a real-time fluorescence quantitative PCR (qPCR), virus isolation, enzyme-linked immunosorbent assay (ELISA), and so on. Some studies have combined viral isolation with the PCR method [22], which can improve the sensitivity of detection. However, it requires cell culture and must be carried out in a special laboratory, which is time consuming and laborious. The ELISA method operation requires expertise, and has disadvantages of its dependence on antibodies and less sensitivity [23]. The qPCR method still requires expertise in operations and instruments, and although it has merits of high sensitivity and specificity [24], it is not suitable for rapid clinical field detection. Therefore, it is particularly important to establish an accurate, specific, and visualized FCV rapid detection method. In recent years, the CRISPR/Cas system has been widely used in genome editing or molecular diagnosis due to its ability to cut foreign genomes [25]. The SHERLOCK system is a powerful diagnostic tool based on the collateral cleavage activity of Cas13a protein [16]. When the CRISPR/Cas system detects alone, the detection results may be inaccurate due to off-target or identification errors of Cas proteins. Combining the CRISPR/Cas system with isothermal amplification technology can improve the recognition rate of its target genes. As such, the sensitivity and specificity of the CRISPR/Cas system was extremely improved and the risk of an off-target error was significantly reduced [26]. With the development of molecular detection methods, the demand for rapid detection has surged. Meanwhile, there are an increasing number of types of pathogens detected by the CRISPR/Cas13a system combined with isothermal amplification methods. Zhang et al. [27] combined RAA with the CRISPR/Cas13a system to detect avian influenza virus, which provided a new detection method by specific amplification of target gene. Hou et al. [28] used specific primers and crRNA-targeting genes to construct a reaction system combining RPA and the CRISPR/Cas13a, and established a rapid, sensitive, and portable method for Vibrio parahaemolyticus detection. Zhao et al. [29] established a sensitive and rapid method for Toxoplasma gondii detection by combining RAA with the CRISPR/Cas13a.

In this study, the Cas13a-RAA-LFD method was established for the rapid detection of FCV. This method can detect the virus in 40 min, which is less than the other common methods, such as virus isolation (several days to 2 weeks), a PCR (around 7 h), and a LAMP assay (70 min) [30]. By binding Cas13a with crRNA, the targeted cutting ability conferred this method with a strong specificity. The detection sensitivity was extremely improved by the combination of the CRISPR/Cas system and RAA. The limit of detection was 10^0^ copies/μL, which was 10 times higher than that of a qPCR and 10,000 times higher than that of a PCR. Zhou et al. [31] established a RAA-CRISPR/Cas12a-LFS method for FCV detection with 37.5 copies/μL of detection limit. Liu et al. [32] established a SYBR Green I fluorescence quantitative RT-PCR method for rapid FCV detection with 10^1^ copies/μL of the limit of detection, but its operation and instruments require expertise and are expensive. The Cas13a-RAA-LFD method in this study has good stability and a high coincidence rate compared with a PCR for clinical detection.

## 5. Conclusions

This study established the Cas13a-RAA-LFD method for FCV rapid detection, with the merits of simplicity, sensitivity, and accuracy. It provides dependable technical support for virus detection in the early stages, helping to control and prevent the spread of FCV.

## Figures and Tables

**Table 1:** Primer and crRNA sequences were utilized in the study.

Name	Sequence (5′-3′)	Location (bp)
FCV-PCR-F	CTGCATTGGAGGGAAACTGT	2282–2301
FCV-PCR-R	ACATCATATGCGGCTCTGAT	2500–2519
FCV-RAA-F1	GTGTGCAATCTGCATTGGGAGTGTGCATGT	2300–2329
FCV-RAA-F2	AGGATCTCACACATCTGTGTCACTTCATAA	2336–2365
FCV-RAA-F3	TGACAGTGTAAGATTGGATGAACTACCCGC	2382–2411
FCV-RAA-R1	AAACTGCCCTGCCAGCGTAAGGTGACGACG	2464–2493
FCV-RAA-R2	TGTCAGGGGCAGTAAGCACATCATATGCGG	2507–2536
FCV-RAA-R3	CTCATCCATCCAGTGTCGCAACATTGCAGG	2542–2571
T7-FCV-RAA-F3	GAAATTAATACGACTCACTATAGGG^1^TGACAGTGTAAGATTGGATGAACTACCCGC	2382–2411
crRNA-IVT	TAATTCGGTGTTTGATTTGGCCTGGGCTGTTTTAGTCCCCTTCGTTTTTGGGGTAGTCTAAATC^2^CCCTATAGTGAGTCGTATTAATTTC	2433–2460
FCV-crRNA	GAUUUAGACUACCCCAAAAACGAAGGGGACUAAAACAGCCCAGGCCAAAUCAAACACCGAAUUA	2433–2460
T7 promoter	GAAATTAATACGACTCACTATAGGG	—

^1^__: T7 promoter sequence and its reverse complementary sequence, ^2^ ﹍: neck ring structure and its reverse complementary sequence.

**Table 2:** Clinical test results of the two methods.

Detection Method	Positive (Copy)	Negative (Copy)	Positive Rate (%)	Coincidence Rate (%)
PCR	43	40	51.81	96.39
Cas13a-RAA-LFD	46	37	55.42	

## Data Availability

The original contributions presented in the study are included in the article, further inquiries can be directed to the corresponding authors.
